# Community-based mental health interventions in low- and middle-income countries: a qualitative study with international experts

**DOI:** 10.1186/s12939-024-02106-6

**Published:** 2024-02-02

**Authors:** Clarissa Giebel, Mark Gabbay, Nipun Shrestha, Gabriel Saldarriaga, Siobhan Reilly, Ross White, Ginger Liu, Dawn Allen, Maria Isabel Zuluaga

**Affiliations:** 1 Department of Primary Care & Mental Health, University of Liverpool, Liverpool, UK; 2 NIHR Applied Research Collaboration North West Coast (ARC NWC), Liverpool, UK; 3 Trials Centre, NHMRC Clinical, University of Sydney, Sydney, Australia; 4 Public Health Faculty, Universidad de Antioquia, Medellin, Colombia; 5 Centre for Applied Dementia Studies, Faculty of Health Studies, University of Bradford, Bradford, UK; 6 School of Psychology, Queen’s University Belfast, Belfast, UK

**Keywords:** Mental health, Interventions, Older adults, Global mental health

## Abstract

**Background:**

Mental health services are scarce in low- and middle-income countries (LMICs), and designing and implementing effective interventions can be difficult. The aim of this international study was to explore the key lessons for developing, implementing, and evaluating community-based mental health and well-being interventions in LMICs, with an additional focus on older adults.

**Methods:**

Research and clinical experts in developing and implementing psychosocial community-based interventions in LMICs were interviewed remotely between October 2021 and January 2022. Participants were recruited via existing global health networks and via published literature searches. Participants were asked about their experiences of developing and implementing interventions, and about key barriers and facilitators during the process. Interviews lasted up to 45 min, and data were analysed using combined inductive and deductive thematic analysis.

**Results:**

Sixteen global mental health experts participated. Five themes with different sub-themes were generated: Mechanisms and contexts; Barriers; Facilitators; Public and stakeholder involvement; Looking through an ageing lens. The development and delivery of mental health interventions in LMICs are facilitated through integration into existing health infrastructures and working with existing job roles as delivery agents. Public and stakeholder involvement are crucial at all stages of development through to implementation to focus on meeting local needs and sustaining participant motivation. Logistical barriers of transport, resources, and location need to be addressed, emphasising local sustainability.

**Conclusions:**

This study provides important insights for how the development, implementation, and evaluation of community-based mental health and well-being interventions in LMICs can be optimised, and can complement general guidance into complex interventions developments.

## Introduction

Experiencing mental health problems such as depression or anxiety in a low- or middle-income country (LMIC) can be particularly challenging. LMICs often lack the necessary mental health infrastructure to support those needs, with LMICs having lower resources (both financial and human) compared to high-income countries. LMIC Governments spend the lowest percentage budget on mental health services compared to high-income countries [1]. The majority of global mental health burden is situated within LMICs, but the investment in and delivery of services to those with mental health problems are mainly inversely proportional to need, due to resource and practical barriers. Research investment in solutions to this are only recently seeking to address this, particularly among older adults.

Older adults (aged 60 + years) residing in LMICs in particular are some of the most vulnerable groups which can be affected by mental health problems. Belonging to the poorest part of the population across different LMICs [2], older adults are more likely to suffer from poor mental health. Findings from the World Health Organisation study on Global Ageing and Adult Health have highlighted how lower socioeconomic background, illiteracy, and female gender are positively associated with geriatric depression [3]. To combat poor mental health and well-being in older adults, a recent systematic review and meta-analysis has highlighted a number of different types of community-based interventions [4]. These include established forms of psychological therapy, exercise, education, social engagement, and multi-component interventions. No single type of intervention was found to be most effective, whilst many were reported to reduce anxiety and depression, and/or improve well-being. Furthermore, as life expectancies increase, the risk of developing dementia before death is also increasing worldwide, including in LMICs, adding to the burden and complexity of meeting care needs.

Given the lack of adequate mental health infrastructure in LMICs, psychosocial interventions are vital to improve the mental health and well-being of underserved populations. Implementing effective, and ideally sustainable, interventions however can be subject to many barriers in different LMIC settings. A recent systematic review by Le et al. [5] has highlighted a number of barriers for wider mental health intervention implementation in LMICs, including community barriers of stigma, client characteristics, as well as structural barriers, such as the wider organisational infrastructure surrounding mental health care provision. The cultural stigma of mental health is a particularly pronounced barrier to accessing care, with mental health problems often hidden within families and communities for fears of being ostracised [1]. Scaling up interventions increases challenges in LMIC environments [6]. Existing evidence has primarily explored the implementation of interventions but a specific focus on effective facilitators remains under-explored.

There is no intervention that works the same in every single context – mental health and well-being interventions need to be adapted to the local populations. One way to achieve this is by involving members of the public and local stakeholders in the development process, as also outlined in the Medical Research Council (UK) guide for Complex Intervention development [7]. The body of research focusing on public involvement in health service development or research in LMICs is limited. Cook et al. [8] reported on the limited availability of public involvement in health research generally, but even less seems to have been published about public involvement in mental health research in LMICs, which showcases a wider lack of reporting on such involvement. This does not suggest such public involvement is not happening, but research needs to report the extent and impact of the public contribution where it is.

Considering the need for improving understanding about barriers and facilitators surrounding aspects of mental health interventions in LMICs – from design and conceptualisation through to implementation and longevity – particularly for older adults, this study involved in-depth interviews with experts in the field gathering their experiences and key lessons of good practice. Specifically, the aim of this qualitative interview study was to explore the experiences and key lessons of developing and implementing community-based mental health interventions for in LMICs, with a particular emphasis drawing upon their expertise directly, or particularly applicable to, interventions for older adults’ mental health needs.

## Methods

### Participants and recruitment

International experts in designing, researching and implementing mental health interventions in LMICs including multidisciplinary academics, analysts and clinicians were eligible to take part. Participants have conducted and published research findings or reports related to mental health interventions in LMICs.

We contacted participants from organizations working on mental health in LMICs, particularly among older adults, building on our research and clinical networks. We also purposefully screened through publication databases for publications on mental health interventions which were published in the past 10 years, and proactively contacted the authors. We obtained ethical approval through the University of Liverpool prior to study commencement [ID: 10216].

### Data and data collection

Participants were interviewed via Zoom between October 2021 and January 2022 using a co-produced semi-structured topic guide. The topic guide was co-developed with public advisors, clinicians and academics. Interviews were conducted by NS and CG; both were experienced in conducting qualitative research. At the beginning of the interview, the researcher took verbal informed consent which was audio recorded. Participants were asked to share their experiences in designing and implementing mental health interventions. Specific emphasis was placed upon how people were involved in designing the interventions; the facilitators and barriers that they had encountered in developing and implementing an effective intervention in the community to support mental health; and the inclusion of, or their views on the relevance for, older adults. Interviews lasted up to 45 min. We also collected background data on participant age, gender, and duration of their experience in mental health.

### Data analysis

All interviews were transcribed into verbatim transcripts. These were anonymised and assessed for accuracy by cross-checking with the audio-recordings. Data were analysed using both inductive and deductive thematic analysis (Braun and Clarke, [9]). Two authors (CG and NS) authors coded each transcript manually line-by-line and generated codes. The first four transcripts were coded using inductive thematic analysis and thus identified codes were discussed between the authors to develop an initial codebook based on consensus. The remaining transcripts were coded through a mix of a deductive and inductive approach. The final list of codes was organised to generate themes which were then discussed with the team members to reach consensus on final themes and their interpretations.

### Public involvement

Two unpaid carers in the UK were involved in developing the topic guide and contributed to manuscript development at all stages, providing a non-academic lens to data analysis and interpretation. Both have been previous carers for a family member living with dementia, and one has been caring for an older relative with mental health problems. They provided an understanding of caring for older adults, including potential mental health needs. Public advisers were recruited via the NIHR Applied Research Collaboration North West Coast, and were reimbursed according to NIHR guidance.

## Results

### Participants

A total of 16 experts took part in this study. Participants were predominantly female (62.5%, *n* = 10), and on average 45 (± 12) years old. People had on average 18 (± 11) years of experience in the global mental health research sector, with professional backgrounds including psychiatry (*n* = 5) and psychology (*n* = 5), as well as pharmacy, preventive medicine, nursing, physical therapy, and global mental health.

### Qualitative findings

Using thematic analysis, we identified four overarching themes and relevant subthemes: Mechanisms and context; Barriers, Facilitators; Public and stakeholder involvement; Looking through an ageing lens. Table 1 includes the quotes for each theme and subtheme, and the following section summarises the themes and subthemes.

**Table 1:** Quotes by themes and sub-themes

Mechanisms and context	
* Highly varied and need for cultural adaptations based on local infrastructure and population needs*	*“from my experience of researching trauma and PTSD it’s an extremely culturally dependant phenomenon and a culturally dependant condition, it doesn’t look the same everywhere.” ****ID14***
	*“So it wasn’t poverty reduction wasn’t a direct goal of the treatment, we didn’t have like a poverty reduction module. But we sort of indirectly ended up working on that and increasing people’s household incomes as a result of using the problem solving therapy module.” ****ID13***
	*“Because you can develop a superb intervention but there is no one to deliver it in the context in which you have developed it then again it won’t work.“**** ID8***
	*“Nepal had a much more sort of community-based activity, in Ethiopia we tried to use existing (10.04) of people employed by the Government in the government sector. Whereas the Nepal group you know they came from a more non-governmental organisation sort of perspective. So, they employed people and got them doing psychological and psychosocial interventions in the community.” ****ID4***
* Delivery agents*	*“I think generally we’ve always stuck to between 6 to 8 sessions so it’s reducing the length of the sessions and then also task shifting treatment delivery so not enough mental health specialists like psychologists and psychiatrist available to implement. So, identifying you know a pair of professionals to do that and that’s, that has also been different across the different studies.” ****ID13***
	*“I think you know there is no best delivery agent and I think it really depends always on the context and I think you need to make decisions based on the context.“ ****ID9***
	*“the CHWs in our setting in our organisation they’ve been equipped to use mobile phones to provide care so it was slightly easier for us because we could integrate the concept of MI into an app and hand it over to the CHWs.” ****ID5***
Barriers	
* Cultural barriers and mental health stigma*	*“I think it just varies by culture or context it’s just again the language that people use around mental health. The kind of explanatory frameworks that people use for why people may be psychologically distressed.“ ****ID9***
	*“I found things like in India you don’t have the vocabulary to describe a lot of emotions like in English you have so many different words to describe just the concept of sadness, you have grief, you have helplessness, you have loss, just sadness, you have depression.” ****ID14***
	*“I would go house to house and try to find out the people with dementia and the family would tell me, no no no no one else lives in this house but the neighbour would tell me that there is someone who is there in the backside of the house and kept on the floor. […]. families were developing their own techniques on how to look after somebody that they didn’t know what was happening to the person.” ****ID8***
	*“in that study we found that even mental even health care providers did not have a lot of training in mental health”**** ID5***
* Logistical barriers*	*“I know it’s frustrating for aid workers or humanitarians, global health practitioners to work with Governments at all levels of local through to federal and very frustrating at times and depending on where you’re trying to intervene there’s a fair amount of corruption.”**** ID3***
	*“you may have the best of intentions, you may have the best of protocol, you may have the best of ideas but this gets stuck there because of the research environment is not there, administration is not really geared up for supporting research.“ ****ID8***
	*“For example, transport issues—if the service user can’t easily travel to the intervention or if there are any physical barriers hindering the service user to come to the intervention.” ****ID10***
	*“we need some kind of logistical support, like in my case for example the venue for the social engagement is very important. So the Office of the Senior Citizen’s Affairs offered their largest room for us to be able to hold the social engagement.“ ****ID1***
* Lack of Resources*	*“if you want to be able to reach and be accessible and affordable to everyone you should be able to subside costs for people that don’t have the same resources as someone else****.” ID14***
* Unavailability of Family carers*	*“there may also be the need to have a sort of like a support network available that can support carers for example.” ****ID8*** *“key is always you know and I think that’s important for older people is also involving the family erm and because you know I see you know older people in low and middle income countries as a as a system. So they live in a family and we are talking about it’s a we are talking about you know a system we are actually targeting. “ ****ID10***
Facilitators	
* Integration into existing health services*	*“in my opinion and in an ideal world implementation work is where we go right away right. So it wouldn’t be about hiring our own interventionist it would be using whoever is in the clinic or whoever is in the community setting.”**** ID13***
	*“ever since we have been using the same video lecture to continuously train and re-train primary care providers who don’t have any mental health training and the video lectures are also available on YouTube for anybody who wants to access it.” ****ID5***
* Personal motivation*	*“the reason why it was well embraced by the elderly people because they can relate to it, they can know the significance of it and they know that it can bring about something good to them and they feel very happy you know being doing the intervention.”**** ID1***
	*“It wasn’t sort of tailored, it wasn’t like a participant came and said I would like to have this or this. But each individual module was tailored to the participant. So with problem solving therapy the participant identifies what are the problems that I want to work on and behavioural activation you go through and you identify what are the person’s values, which they tell you. It’s very much the participant themselves that identifies what matters to them. But the modules themselves were predefined, predetermined.”**** ID13***
* Continuous engagement between research lead and delivery agents on the ground*	*“having 1 or 2 key persons to bridge the gap between the programme team and the research team and have frequent communications I think that’s equally important.“ ****ID5***
Public and stakeholder involvement	
* Adapting to local needs*	*“co-designing a mental health intervention requires an interdisciplinary approach. So you really cannot do it by yourself” ****ID1***
	*“the best way to do it is to co-create you know to have recipients of the care or intervention be part of the framing and you know I think we really try to do that as much as possible and we always have. I mean from a disability perspective there’s a very strong approach to sort of the concept of nothing about us without us. So it’s always been co-created but that doesn’t mean it’s effective and there’s always gaps that still happen. So co-design is the right way to do it it’s lengthy it takes a lot longer but it creates a better product and a more effective product at the end.” ****ID3***
	*“before you begin your research you have to coordinate with the community. I mean you need to talk to made the city health officer, the senior citizen organisation, even the city mayor you know you have to communicate with them and then the ethics committee required me to submit like a memoranda of understanding since I am about to do an intervention.” ****ID1***
	*“after a few discussions we had conducted workshops where we would talk about the framework and what is the end goal and then we met regularly for I would say next 3 to 4 months err where we shared the updates and like wherever the design team was, co-design team in in terms of the app development and we would conduct meetings and then take the feedback and then go back to the development team and take it based on the feedback and then come to the CHWs and do that over and over again until was ready to be tested out.” ****ID5***
* Continued involvement*	*“and then also we’ve kind of expanded which we didn’t in catalyst but we found in addition to the Ministry of Health or the other Government Ministries and the policy makers, if we’re trying to think about sustainability. We need to get their buy in because that’s kind of sets the agenda and also the financial planning within the country but we also want kind of what we call community influencers. So who are the people within the community who if they say this is a great thing and we think this will be very helpful to us people will follow suit. So that might be pharmacists, religious leaders, traditional healers within the village, whoever is like the kind of the head of the village or the chief.”**** ID2***
	*“I think the thing that really stands out is the importance of involving local stakeholders in the provision of care so (07.37) it’s not about just transplanting an existing intervention.” ****ID14***
	*“that was useful input so all of these different range of community stakeholders were involved and as I say more at the beginning the caregivers of people with mental health conditions were involved and as time went on we were also able to involve people with lived experience as well.” ****ID4***
* Community ownership*	*“so basically the interventions are not my own decisions. I mean of course my intervention is justified with the literature but the components of the interventions are actually coming from the voices of the elderly and the head of the Office for the Senior Citizens Affairs.“ ****ID1***
	*“I think that engaging key stakeholders is a must, all stakeholders, both from the supply side, so these are the people are going to be delivering the intervention or the organisations involved in delivery.“ ****ID8***
	*“Because in the end we want to develop a sustainable intervention and we want to make sure that the intervention still exists after the research has ended and the money has run out.” ****ID9***
Looking through an ageing lens	*“But I think the challenges to working with older adults is going to be slightly different I would say because the issues of access, the issues of logistic and if they are able to receive the intervention or understand the intervention or consent to participation or not. Or even if you are using some digital technology how, how good are they to use those technologies. I think those sort of things would be different but yes I think there would also be a lot of similarities though” ****ID5***
	*“there may also be the need to have a sort of like a support network available that can support carers for example.“**** ID8***
	*“I think that’s important for older people is also involving the family and because I see older people in low and middle-income countries as a system. So they live in a family and we are talking about it’s a system we are actually targeting. So I think we also have to address the needs of the families and support the families in helping to deal with the problem that the older person might have and often they are also stressed and drained.“**** ID10***

### Theme: Mechanisms and context

#### Highly varied and need for cultural adaptations based on local infrastructure and population needs

Interventions need to be adapted to the local needs of populations and the existing infrastructure. This context varies across countries and across different regions within countries, so that in-depth groundwork and public and stakeholder involvement needs to support the shaping of the intervention to the local context. This generates a complex overall intervention where multiple countries are involved, as undertaking groundwork and stakeholder involvement in each country can lead variations in design and implementation elements.

One particular example of adapting interventions to localised contexts was provided by a researcher having conducted interventions in South Africa. Lack of income and associated poverty was a major issue for the target population, so a module providing support to improve household income was added to the core intervention aimed at reducing depression, as a basic pre-requisite for effectiveness.

Working closely with the local existing infrastructure is key to intervention implementation success, an issue that also emerged as a key element in the ‘Facilitators’ theme. This involves not only working with existing health care systems, and on-site care professionals, but also the local research and community infrastructure. Certain communities will have different community leaders, high status and power attributed to local religious leaders, or mayors for example. For interventions to be effectively implemented, they need to be embedded into these local infrastructures as opposed to creating something completely new. This embedding can then facilitate implementation, and likely success in uptake and outcome.

#### Delivery agents

One component which also needs to be adjusted for an intervention in a different country or setting is the person/group of people delivering the intervention – the delivery agent(s). As highlighted, integrating the intervention into the existing health care infrastructure is strongly recommended by experts. This means to work with different roles in delivering an intervention, as a health care professional in one country may not have the same role or connections with the local community as in another country. The type of delivery agent also depends on availability within each setting.

One role which emerged repeatedly across the interviews was that of the Community Health Worker (CHW). CHWs were frequently employed as delivery agents of psychosocial community interventions in LMICs, amongst others due to their good connectivity with the populations.

### Theme: Barriers

A number of barriers were raised by experts, some of which are included under the Facilitators theme as participants focused on the solutions rather than the challenges (e.g. ensuring integration into existing health services, need for community involvement). These included stigma and lack of information about mental health and associated burden in LMICs, cultural barriers to accessing care, intervention logistics.

#### Cultural barriers and mental health stigma

In many LMICs, the language to express mental health problems is not readily available. Often, the stigma surrounding mental health is so great that people hide their relatives with a known mental health problem or condition which they cannot easily explain, such as dementia. Some experts who worked in the field of dementia, reported how families hid their relatives with the condition away from the outside world, feeling too embarrassed and fearful of associated stigma.

Not acknowledging poor mental health or deteriorating cognitive conditions such as dementia can be a substantial barrier in getting help and support in the first place. Interventions may be meeting the needs of the local population, but if people are not willing to engage for fear of wider stigma, then the intervention is not going to be acceptable or successful.

Similarly, some care professionals working within those setting have been reported to have limited knowledge about, or skills in mental health problems and care. This is a barrier to providing adequate mental health care in the first place, or recognising it as a priority for service delivery.

#### Logistical barriers

Many different logistical barriers were noted by experts. These could broadly be categorised into barriers of implementing the intervention, and barriers for service users in accessing the intervention. Concerning the former, some experts raised issues with involving local Governments, as these could be corrupt and difficult to deal with. Whilst many experts reported on the benefits of involving local mayors for example to raise the legitimacy of the intervention within the local community, many experts cited examples of corrupt officials from local through to federal government.

Logistical barriers in implementing an intervention can also include a lack of existing research infrastructure. An intervention may meet the needs of the local population and be desirable, but with the research infrastructure not set up for this in an LMIC, it is impossible to evaluate the process and impact of implementing the intervention. This has been raised by a number of experts who have been working on different interventions over the years.

In terms of accessing the intervention, there are multiple logistical barriers which need to be considered. Transport has been raised by many experts, as many service users in poorer communities may not have sufficient funds to pay for or use their own transport to access the programme/care being offered. This consequently impacts on decisions about the setting and locality of the intervention. A related consideration is the actual building (if it is set in a specific place). Religious buildings should be avoided, as some participants may not be religious or have different religious beliefs, and an intervention should be neutral and accessible to anyone within the target population.

#### Lack of resources

Lacking the required financial resources to attend the intervention can also be a common barrier for people in LMICs. As indicated in one study for example, an intervention module was created to improve household income (see 1. Facilitators – personal motivation). Therefore, to ensure equitable uptake of the intervention for everyone, the costs to attending the intervention should be subsidised for those in greater financial need. This may involve providing free transport to and from the intervention.

#### Unavailability of family carers

For some target populations, specifically older adults, family carers are often a vital part of their support network. More vulnerable populations may therefore rely on their family carer to support them in accessing the intervention. This can often be a barrier, as families may have work or other caring commitments, and can be unavailable to support their relative to attend every single intervention session. This needs to be taken into account to enable easier access of the intervention to all population groups, by scheduling interventions at suitable times of the day and taking away the need for family carers having to accompany their relative to an intervention for example, such as via providing transport or other personal support.

### Theme: Facilitators

A large number of facilitators to effective intervention development, implementation, and outcomes were mentioned by experts. Public and stakeholder involvement was identified as a key facilitator, but given the large amount of experiences and nuances in involvement expressed, public and stakeholder involvement is a separate theme.

#### Integration into existing health services

One way to help improve the sustainability of the intervention, but also its immediate delivery, is by integrating the intervention into the existing health service infrastructure, according to experts. This involves engaging lower-skilled, local and existing staff to deliver the interventions, with few high-skilled staff available. One such role could be Community Health Workers (CHWs), an approach used in many experts’ studies. Integrating interventions into existing infrastructures can also provide fiscal advantages by utilising existing assets and resources.

The advantage of working with existing staff and providing training to them is that the local community already know the staff and thus more likely to trust and engage in the intervention. Vice versa, staff know the local population, and may know specific individuals who would benefit from participating in the intervention. This rapport can otherwise take a long time to build up, particularly with a new type of treatment (the intervention), which the potential participant group has not engaged with before. Engaging existing health care professionals thus allow easier acceptance, as well as delivery, of the intervention.

An added advantage of working with existing health care professionals is the opportunity to provide mental health training and general awareness of mental health in the existing workforce. These resources can then also be employed to train other health care staff who are not part of the intervention, creating increased workforce capacity.

#### Personal motivation

To ensure continued uptake of the intervention and reduced drop-out rates, experts highlighted the importance of addressing the personal motivation for intervention participants. The intervention not only needs to meet the needs of the local population, but also needs to be tailored to the wishes of the target population – providing agency and choice for those receiving the intervention. This includes understanding these motivations both during public and stakeholder involvement in the development process and co-developing the intervention so that motivations for getting more socially engaged, improving one’s well-being, or simply taking part in a community-owned activity are understood and enhanced.

Shaping interventions to meet the personal needs and motivations of participants can also involve providing different options or modules to participate in. As one expert highlights, modules can be pre-defined (through stakeholder involvement), yet the actual intervention components that someone participates in can be self-selected, increasing autonomy.

#### Continuous engagement between research lead and delivery agents on the ground

Some experts highlighted a need for good and continuous communication between the research team, which often is not fully based in a specific country, the programme team in the country, as well as the delivery agents. Continuous monitoring of the intervention by having frequent meetings with the intervention deliverers can help to ensure that a consistent intervention approach is used in practice. Continuous monitoring can further help to highlight potential issues or concerns when conducting the intervention. This clear communication channel can help facilitate the delivery, and potential success, of the intervention.

### Theme: Public and stakeholder involvement

#### Adapting to local needs

Public and stakeholder involvement was considered a key facilitator and necessity in the development of a culturally adapted, meaningful, engaging, and well attended psychosocial community intervention. Whilst it is a facilitator as well, it was considered so crucial by all interviewed experts that it was generated into its own standalone theme.

Each expert shared a great deal about their public and stakeholder involvement across individual and multi-country research. Community-based interventions can only be developed and implemented via multi-stakeholder involvement. Experts stated how implementing an established intervention, such as Cognitive Behavioural Therapy, in an LMIC without any adaptations to local needs would have a low probability of success.

The first step of public and stakeholder involvement is thus assessing the needs of the local community and population. This can be done by holding different workshops at the beginning of the intervention development, which can be complemented by research into the needs of the local population. Involvement needs to be as inclusive as possible, by not only involving the targeted population (such as older adults in a region/ country), but also care professionals and local decision-makers (religious and community leaders, local government, mayors). This allows a broad view on the needs and wishes of the target population, whilst similarly allowing key delivery agents to help shape to delivery mode of the intervention. The involvement of local decision-makers from the very beginning is also key in generating ‘buy-in’ for the intervention among the local population.

#### Continued involvement

Throughout the intervention, these representative groups ought to continue to be involved, to ensure that the intervention meets the needs of the local population. Stakeholders can also be themselves involved in delivering the care, which further raises the integration of public and stakeholder involvement in the overall intervention process.

Another important stakeholder group identified was the immediate family or carers of participants. This is particularly the case for older or more vulnerable adults as intervention recipients.

#### Community ownership

Having Community Champions who are key advocates for the intervention is key to its success in terms of uptake and often outcome. This can be achieved by having community leaders and stakeholders, including local Governments, mayors, or religious leaders, involved from the beginning. This involvement raises the acceptability of the intervention in the eyes of the local community/communities, as it is not outside experts who are entering the community delivering something new and unknown, but the community has an active part in the development, and subsequent ownership of the intervention.

This ownership is crucial also for the sustainability of the intervention beyond its funded lifespan. If the community is championing the intervention, they are more likely to find ways in which to integrate the intervention subsequently into everyday local life.

### Theme: Looking through an ageing lens

Interventions specifically targeting older adults tend to require additional adjustments. People may be frail and in greater need of physical support to reach the intervention, needing more frequent breaks or shorter sessions. The potential frailty and wider comorbidities also raises a potential need for addressing not only the mental health and well-being of participants, but also their physical health.

Many older adults may also rely on family in their day-to-day life more than someone of working age. This can cause severe burden on the well-being of the family carer(s), suggesting that carers and their wider support network should be actively integrated into the intervention and provided with their own support component. In addition, carers need to be involved in developing interventions and thus be part of the public engagement.

## Discussion

Our background searches suggest this is the first study to have explored the key lessons for the effective development, implementation, and evaluation of community-based mental health and well-being interventions in LMICs with a specific focus on older adults. Whilst the effective integration of interventions can be hindered by a myriad of barriers, experts have highlighted key facilitators to ultimately enable improved mental health for LMIC populations.

Public and stakeholder involvement was raised as a crucial component of every aspect of the intervention process. Integrating large elements of public and stakeholder involvement is not only a facilitator, but is vital for any such mental health intervention to take place. Participants reported on different ways to engage those groups in receipt of the intervention, those delivering an intervention, and the wider community. Considering that LMIC characteristics, cultures, and environments vary considerably, with different health, political, and social infrastructures, it is essential to first establish the needs of local populations and involve delivery agents and the community in the development of the intervention, to achieve community buy-in. Public and stakeholder involvement is vital, and includes involving people with lived experiences and service users, such as older adults and their family carers, which helps to embed the personal lived experiences of people in the research, and to support buy-in from local communities, and may become sustainable beyond the project. Public and stakeholder involvement is a key component of UK-based research, such as in ageing and dementia [4], whilst evidence from LMICs is only more recently starting to grow. Ryan et al. [10] reported burgeoning efforts of public and stakeholder involvement, with limited evidence on the impacts of service user involvement in global mental health research reported to date.

With high levels of mental health stigma [1], highlighted as a common barrier in our study, having Community Champions and local leaders (whether religious, political, or general community) outwardly supporting an intervention to address mental health and well-being, serves as a key enabler to generate trust from potential participants. This is corroborated in a meta-review by Kohrt et al. [11], which highlights the role of communities in wider mental health care, as opposed to psychosocial interventions, in LMICs. Importantly, this community ownership increases the chances of the sustainability of the intervention, with various stakeholders involved and invested from the start. Further support for the value of community participation was evidenced by Murphy et al. [12], reporting also on contextual considerations, stigma, and resources as additional barriers to successful global mental health projects, experienced by 29 recipients of Canadian global mental health funding. Thus, implementing temporarily-funded mental health interventions in LMICs using in-depth involvement can have long-lasting benefits to improve mental health service provision in these highly underserved regions.

Increased sustainability (allied to the UN sustainability goals, as well as improved intervention delivery, could also be achieved by working within the existing health infrastructure. By integrating an intervention, such as problem-solving therapy, into an existing infrastructure which is known to the local population, participants are more likely to engage in the intervention. Where an intervention is completely separately from any existing systems, people are less likely to engage, and the intervention will more likely disappear once the funding ceases. This can pose many benefits not only to the served population, but also to the existing health care staff. Staff can receive training as part of the intervention, which can be passed on. Considering the limited mental health awareness not only in the general population [13] but also in healthcare staff in LMICs [14, 15], embedding such interventions into existing infrastructure can generate wider mental health awareness, thereby potentially breaking down another key identified barrier in uptake – stigma [1]. This could also link to greater awareness of dementia, by potentially generating wider mental health awareness for family carers.

Working with existing health care professionals or staff working within the health care infrastructure is another key aspect of improved delivery modes. One role which featured especially was that of the Community Health Worker (CHW). CHWs are non-medically trained members from the community whose role it is to promote health and deliver limited health support [16]. Being a member of the community allows them easier access to community members. There are insufficient medically-trained health and mental health professionals in LMICs, so that having trained lay workers educating about health and mental health and providing therapy after having received training has proven to be an efficient method [17]. This task-sharing approach has been found to improve mental health care in rural and other low-resource settings via CHWs [18]. Whilst lay health workers, such as CHWs, are therefore a highly advantageous solution to tackling poor health and mental health in LMICs, there is a risk of overloading these lay professionals with too many tasks without providing adequate support, training, and continued supervision [19]. When considering the integration of psychosocial interventions in the community therefore, the intervention itself needs to factor in improved training and guidance for CHWs, if these are the delivery agents, or share intervention delivery across different lay and medically-trained health workers.

When delivering an intervention, the focus not only needs to be on the service user and the delivery agents, but also on the wider support network of the participant. This was particularly raised for older adults, who are frequently having a family carer or are supported by their wider family. Providing unpaid care for an older relative can be demanding on the mental health of carers as well (van den [20], which has been particularly exacerbated during the COVID-19 pandemic [21]. Support for unpaid carers is often inadequate, and carers face many unmet needs themselves [22, 23]. Therefore, psychosocial interventions should provide an add-on for family carers to support them as individuals, which in turn also supports the service user to a greater extent.

Unpaid carers were also referred to as enablers of interventions. Particularly for older adults who are more likely to be frail and suffer from comorbidities, getting practical support to access an intervention is important. As highlighted, this may include offering free transport for example. An unpaid carer can also help facilitate, or hinder, accessing the intervention though – either face-to-face or digitally. Where unpaid carers are unavailable due to other commitments, including work and caring duties, older adults can struggle in accessing an intervention. In an increased digital world since the pandemic, providing digital interventions may be a solution to providing access where unpaid carers are unavailable. However, considering the generally high digital divide [24], with many older adults experiencing reduced digital literacy compared to younger generations, especially older adults living with dementia [25, 26], and often poor access to digital technology in LMICs [27], interventions ought to be delivered face-to-face wherever possible by addressing logistical barriers instead.

Whilst this study benefits from a diverse range of expertise on different types of psychosocial interventions and across different LMICs, the study has some limitations. Findings are purely based on academic and clinical global mental health experts. We did not interview delivery agents or service users. The reason for this was that the academic and clinical experts will have an overarching point of view of the intervention stages, having managed or been involved in intervention development, early implementation and research. However, it is important to capture the experiences of those providing and receiving the intervention also, which to date appears not to have been evidenced in detail. [28, 29]

## Conclusions

This study provides guidance on more effective development, implementation, and evaluation of community-based psychosocial interventions in LMICs. With limited mental health resources, interventions should be integrated into existing health infrastructures and engage local delivery agents, to improve the reach of the intervention, training surrounding mental health in the health care workforce, and improve the potential for sustainability. When conducting global mental health research with older adults, a particular focus needs to be paid to the families caring for their older relatives, both within the public engagement aspect and the delivery of an intervention. Only when fully engaged with the local population and stakeholder groups can an intervention address participants’ needs, which for multi-country interventions means multi-country public and stakeholder involvement and intervention adaptation. The benefits of this embedded involvement reach beyond the life-span of the funded intervention and can engage strong Community ownership, enabling its sustainability further. Future psychosocial interventions in LMICs can take key lessons from this study, which will be of particular use to professionals who have not conducted global mental health interventions previously.

## Data Availability

The datasets generated and/or analysed during the current study are not publicly available due ethics but are available from the corresponding author on reasonable request.
